# Lessons learnt from the first controlled human malaria infection study conducted in Nairobi, Kenya

**DOI:** 10.1186/s12936-015-0671-x

**Published:** 2015-04-28

**Authors:** Susanne H Hodgson, Elizabeth Juma, Amina Salim, Charles Magiri, Daniel Njenga, Sassy Molyneux, Patricia Njuguna, Ken Awuondo, Brett Lowe, Peter F Billingsley, Andrew O Cole, Caroline Ogwang, Faith Osier, Roma Chilengi, Stephen L Hoffman, Simon J Draper, Bernhards Ogutu, Kevin Marsh

**Affiliations:** The Jenner Institute, University of Oxford, Oxford, UK; Centre for Clinical Research, Kenya Medical Research Institute, Nairobi, Kenya; Centre for Research in Therapeutic Sciences, Strathmore University, Nairobi, Kenya; Kenya Medical Research Institute - Wellcome Trust, Centre for Geographical Medical Research (Coast), Kilifi, Kenya; Sanaria Inc, Rockville, MD USA; Centre for Infectious Disease Research in Zambia, Lusaka, Zambia

**Keywords:** CHMI, Kenya, Challenge, PfSPZ Challenge, Lessons, *Plasmodium falciparum*, Vaccine, Drug, African, Malaria

## Abstract

**Background:**

Controlled human malaria infection (CHMI) studies, in which healthy volunteers are infected with *Plasmodium falciparum* to assess the efficacy of novel malaria vaccines and drugs, have become a vital tool to accelerate vaccine and drug development. CHMI studies provide a cost-effective and expeditious way to circumvent the use of large-scale field efficacy studies to deselect intervention candidates. However, to date few modern CHMI studies have been performed in malaria-endemic countries.

**Methods:**

An open-label, randomized pilot CHMI study was conducted using aseptic, purified, cryopreserved, infectious *P. falciparum* sporozoites (SPZ) (Sanaria® PfSPZ Challenge) administered intramuscularly (IM) to healthy Kenyan adults (n = 28) with varying degrees of prior exposure to *P. falciparum*. The purpose of the study was to establish the PfSPZ Challenge CHMI model in a Kenyan setting with the aim of increasing the international capacity for efficacy testing of malaria vaccines and drugs, and allowing earlier assessment of efficacy in a population for which interventions are being developed. This was part of the EDCTP-funded capacity development of the CHMI platform in Africa.

**Discussion:**

This paper discusses in detail lessons learnt from conducting the first CHMI study in Kenya. Issues pertinent to the African setting, including community sensitization, consent and recruitment are considered. Detailed reasoning regarding the study design (for example, dose and route of administration of PfSPZ Challenge, criteria for grouping volunteers according to prior exposure to malaria and duration of follow-up post CHMI) are given and changes other centres may want to consider for future studies are suggested.

**Conclusions:**

Performing CHMI studies in an African setting presents unique but surmountable challenges and offers great opportunity for acceleration of malaria vaccine and drug development. The reflections in this paper aim to aid other centres and partners intending to use the CHMI model in Africa.

**Electronic supplementary material:**

The online version of this article (doi:10.1186/s12936-015-0671-x) contains supplementary material, which is available to authorized users.

## Background

Controlled human malaria infection (CHMI) studies, in which healthy volunteers are infected with *Plasmodium falciparum* to assess the efficacy of novel malaria vaccines and drugs, have become a vital tool to accelerate vaccine and drug development [1-5]. CHMI studies in malaria-naïve volunteers have been shown to accurately predict vaccine efficacy in the target African paediatric population [6] and provide a cost-effective and expeditious way to circumvent the use of large-scale field efficacy studies to deselect intervention candidates [7,8].

Conducting CHMI trials in malaria-endemic rather than ‘northern’ malaria-naïve countries has a number of key advantages. As well as allowing early assessment of vaccine efficacy in a population with the same genetic background as the eventual target population, there is the opportunity to assess the effect of prior exposure to malaria, and the immunological priming this provides, on vaccine efficacy [8]. Conducting CHMI trials is also important in building the capacity of African research institutions to become involved in the earlier stages of vaccine or drug development. However, to date, CHMI trials have rarely been conducted in malaria-endemic regions, primarily because of the lack of access to the appropriate parasite culture and insectary facilities necessary for mosquito-bite CHMI studies [1,6,8].

The development of aseptic, purified, cryopreserved, infectious *P. falciparum* sporozoites (SPZ) for injection (Sanaria® PfSPZ Challenge) has helped overcome this problem [9]. PfSPZ Challenge is stored in liquid-nitrogen-vapour-phase at known concentrations and as such can be easily transported to sites, allowing administration of a known, predefined number of SPZ, and reduction in trial-to-trial and site-to-site variation in infecting dose [10,11].

To date, three CHMI trials using PfSPZ Challenge have been conducted in malaria-endemic regions: the first in Tanzania [12], the second in Kenya [13] and the third in Gabon (Lell *et al.,* unpublished)*.*, This paper provides a discussion of the experiences and lessons learnt conducting the first CHMI study in Kenya which should be useful given the potential future importance of the African CHMI platform, planned African CHMI studies at new sites (SLH, pers comm) and the unique challenges faced when performing CHMI studies in the developing world.

## Methods

**Figure 1:**
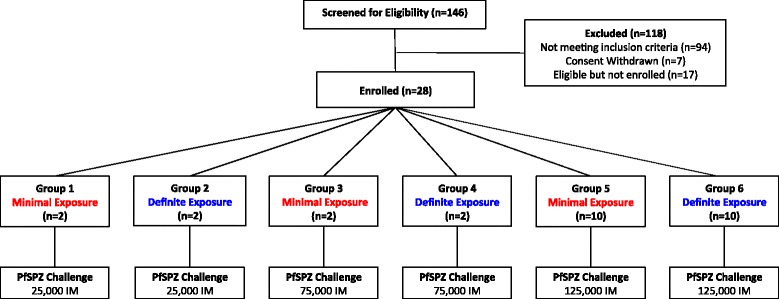
Study design and volunteer recruitment. 118 participants were excluded following screening. For reasons see Figure 2. In each group, the total dose of sporozoites was split between two injection sites and administered as two 50 μL injections, one in each deltoid.

An open-label, randomized, pilot CHMI study was conducted using PfSPZ Challenge administered intramuscularly (IM) to healthy Kenyan adults (n = 28) with varying degrees of prior natural exposure (Figure 1) [13]. The purpose of the study was to establish and assess feasibility of the PfSPZ CHMI model in a Kenyan setting with the aim of increasing the international capacity for efficacy testing of malaria vaccines and drugs, and allowing earlier assessment of efficacy in a population for which vaccines or drugs are being developed.

The study was conducted at the KEMRI Centre for Clinical Research, Nairobi, Kenya according to the principles of the Declaration of Helsinki, in accordance with Good Clinical Practice and in line with WHO guidelines on the conduct of sporozoite CHMI studies [14]. The study was registered with the Pan African Clinical Trial Registry (PACTR20121100033272) and conducted in accordance with an Investigational New Drug (IND) application filed with the US Food and Drug Administration FDA (IND 14267).

Inclusion and exclusion criteria for the study are shown in Additional file 1: Table S1. At screening, in addition to a full medical history, physical examination, urinalysis and pregnancy test in females, safety blood tests (including complete blood count, haemoglobinopathy screen, electrolytes, liver function tests and assays for HIV, hepatitis B, hepatitis C), and an electrocardiogram were performed for each volunteer to identify and exclude any individuals with baseline abnormalities [15,16]. Highly sensitive qPCR for *P. falciparum* was performed on screening blood samples to identify and exclude any individuals with asymptomatic parasitaemia. Volunteers positive for *P. falciparum* by qPCR at screening were treated with a therapeutic course of artemether/lumefantrine (Co-Artem®) as per national guidelines. All volunteers were asked not to leave Nairobi in the four weeks between screening and enrolment in order to prevent any community-acquired *P. falciparum* infection prior to CHMI. Volunteers with clinically significant illness at screening were excluded and referred for appropriate management as per national guidelines.

All volunteers were managed in an in-patient setting from the day before administration of PfSPZ Challenge (C-1) until completion of anti-malarial therapy (maximum 23 days post administration of PfSPZ Challenge). All volunteers were successfully infected with *P. falciparum* as confirmed by qPCR*.* All but one volunteer (110) became blood-film positive prior to day 21 post injection of PfSPZ Challenge (C+21). All volunteers completed anti-malarial therapy and follow-up as scheduled. Adverse events (AEs) associated with clinical malaria infection were broadly similar to those observed in ‘northern’ clinical trials centres [13,17,18].

## Discussion

### Consultation

A detailed plan for community sensitization for the study was drawn up in advance of the study (Table 1). Four years before the start of the trial, consultations began with key stakeholders in Kenya see below. At these multidisciplinary meetings, ethical, logistical, funding, and scientific issues were discussed. All parties supported the establishment of a CHMI centre in Kenya as a key objective in line with national and regional research priorities. A decision was made for Nairobi to be the preferred site for the first CHMI study, given the ease of access to tertiary medical centres, the relative lack of natural malaria transmission and large concentration of educated individuals best placed to provide informed consent. Initial discussions focused on developing an insectary capable of supporting mosquitoes imported from abroad for mosquito-bite CHMI studies [1]. However, when PfSPZ Challenge became available, this option was chosen due to its practical advantages [9].

**Table 1:** **Community sensitization plan**

	**Universities**	**ERC**	**General public**	**Participants**
**Consultation/gatekeeping**	Seek advice from senior managers and request permission to discuss study with students	Discussion regarding suitability of study in Kenya and ethical concerns.	Nil	Nil
		Advised on compensation levels.		
		Approved study.		
**Sensitization**	General meetings outlining role of KEMRI and study	Nil	Nil	General meetings outlining role of KEMRI and study and invitation to attend a more detailed information meeting
**Feedback**	Meeting with University management to feedback results	Written report of study findings, challenges and lessons learnt	Nil	Presentation of study findings in ‘grand round’ setting

ERC = KEMRI Ethics Review Committee.

KEMRI = Kenyan Medical Research Institute.

Kenyan stakeholders consulted in advance of submission of study for ethical and regulatory approval

- Ministry of Health
- Pharmacy and Poisons Board (National Regulatory Agency)
- Kenya Medical Research Institute (KEMRI) Ethics Research
- Committee
- National Commission for Science, Technology and Innovation
- KEMRI
- Consortium for National Health Research Kenya (NGO)
- University of Nairobi Institute of Tropical & Infectious Diseases
- University of Nairobi School of Medicine
- Ministry of Health Division of Vaccines and Immunization

#### Conclusion

Detailed discussion with key stakeholders years ahead of the planned study was an important two-way process, increasing understanding and acceptance of the CHMI model in key national stakeholders and allowing important feedback to guide the study design and increase speed of ethical and regulatory approvals at a later date.

### Ethical and regulatory approvals

Kenya has a multi-tiered system of human subjects research review where proposals go through three or more rounds of review prior to approval. Following review by the KEMRI Kilifi Centre Scientific Committee (CSC) and the KEMRI Centre for Clinical Research CSC, the study was reviewed by the national KEMRI Scientific Steering Committee (SSC) and then later approved by the KEMRI Ethics Review Committee (ERC). The ERC requested additional information regarding access to intensive care facilities, the rationale for the proposed amount of monetary compensation (see Consent) and a clear description of how the target population of educated adults would be approached. Final ethical approval to conduct the study was received six months after initial submission to the CSC.

The University of Oxford requires ethical approval from the Oxford Tropical Research Ethics Committee (OXTREC) for all clinical trials it sponsors overseas. Prior to approval, OXTREC requested justification for the proposed amount of monetary compensation, clarification on consent processes for long-term storage of samples and subsequent assays on these samples and assurances regarding the sensitivity of PfSPZ Challenge to the proposed anti-malarial therapy. Ethics approval from OXTREC took two months from initial submission.

Prior to regulatory approval, the Kenyan Pharmacy and Poisons Board requested assurances as to how PfSPZ Challenge would be transported and stored at -140°C. Regulatory approval took three weeks from initial submission. An import permit was granted for PfSPZ Challenge and the two required diluents for injection (25% human albumin solution and phosphate buffered saline), which were imported from the USA. A test shipment labelled as PfSPZ Challenge and diluents passed through Customs with no delays. PfSPZ Challenge is subject to an Investigational New Drug Application filed with the FDA and sponsored by its manufacturer, Sanaria Inc. In this capacity the FDA reviewed and approved the study.

Just prior to the start of screening, the international CHMI community was made aware of a second cardiac serious AE occurring in a mosquito-bite CHMI study assessing a malaria vaccine at Radboud University Medical Centre, Nijmegen, The Netherlands [16]. The study protocol and consent form was updated to reflect this new information and add an electrocardiogram (ECG) to the investigations performed on all individuals undergoing screening. Approval was sought for these amendments from the relevant ethical and regulatory authorities. All approvals were obtained within 37 days of the applications, allowing the study to go ahead according to the original timelines.

#### Conclusion

All bodies were supportive of the study and recognized its importance. The initial multistage approval process did take a considerable time but ensured the study was rigorously reviewed. Given that the dates of the CHMI aspect of study were required to be set well in advance (see Study design), the swift approval of the substantial amendment was extremely important as it allowed the study to proceed on schedule with appropriate updated information for volunteers.

### Community sensitization

All community sensitization occurred following approval of the study and at a local, rather than national level. Therefore, feedback gained from the community will be used to inform the design of future CHMI studies. Given the novel and complex study nature of the study, [19] community sensitization focused on local medical schools as it was felt this population were most likely to have some existing understanding of the concept of research. Letters were sent to the heads of the three main medical schools in Nairobi, requesting permission to present the study to staff and students. This occurred at three meetings which took place approximately six weeks before the start of screening, and took the form of a formal presentation by the Principal Investigator on the background and purpose of CHMI studies, followed by details of the planned study. Approximately 200 individuals in total attended these meetings. The list of questions raised at the meetings and the reasons attendees gave to participate or not in the study are shown below. About half of individuals at the meetings said they would consider participating in the study. Posters advertising the trial were placed at the medical schools and interested individuals were asked to contact the study team to attend an individual information meeting and receive the volunteer information sheet (VIS; see Additional file 1). Two-hundred and forty-three individuals subsequently received the VIS and 146 attended for screening visits at a later date.

**Questions raised at community sensitization meetings**

*Questions Addressed in the VIS:*

- Does one have to stay in hospital throughout the study?
- What is the compensation that will be given?
- What are the risks of participating?
- If it happens that I do not react as expected, what will happen?
- The method of injection is new - is it safe?
- How do you compare mosquito and injection?
- What will make you choose me to participate in the study?
- Will I be subjected to pricking (blood sampling)?
- What if I have commitments at the time of the study?
- In brief, what message can I give my friends about this study?
- How many people will participate?
- What are your expectations?
- I know my parents will want to know – are there other people involved in the study other than students say for example a doctor or just other people?
- What if I react on day 1?
- With the dates of the study procedures you have given it seems this might interfere with our studies and we all know our education should come first. What do you have to say about this?
- What are the benefits of participation in this study?
- Is there risk of infection?
- Now that we are going to take part in the study who will do the research, KEMRI or the students?
- How much of our time do you need for the study?

*Questions not Addressed in the VIS:*

- Where can I get more information about this product?
- Will you be involving only people from Nairobi or also outside Nairobi?
- Do you just sit down the whole day doing nothing?
- Is visiting allowed?
- Will there be a document to say I have participated in this study, like a certificate or a letter saying I was involved?

**Reasons given at community sensitization meetings to participate or decline to participate in the study**

*Reasons given to participate*

- I am patriotic and I would love to take part in this study to help my country.
- One day I will have my own family and by my participation in this particular study I will be helping to generate very important medical knowledge that will one day help my own children and other families in Kenya.
- For curiosity.
- I would have been happy to volunteer in the malaria vaccine study (RTS,S) if given the opportunity as I have seen it has generated very useful data.

*Reasons given not to participate*

- I am scared of my parents’ reaction.
- I will be having job interviews during the time.
- I am scared of needles.
- Staying away for that long and being unable to do my things.
- It is a new thing – we do not know what to expect.

#### Conclusion

Meetings at the medical schools provided an excellent forum to explain the study to a target, educated audience with a prior understanding of the concept of research and discuss and explain all aspects of the study. The VIS of future studies will be updated to address those questions raised at community sensitisation meetings that were not addressed in the VIS.

### Consent

#### Informed consent

At screening, at least 24 hours after individuals received the VIS, volunteers discussed the study in detail in a one-on-one setting with Kenyan study investigators who were also physicians. All volunteers who presented for screening were happy to consent to participate in the study. Prior to obtaining written consent, all volunteers were required to undertake a written questionnaire to assess their understanding of the study (Additional file 1). Volunteers were required to answer all questions correctly before being allowed to consent to the study and were only allowed to attempt the questionnaire a maximum of three times. After each attempt, the participant was allowed to ask for clarification of any points from the study investigator. Of the 146 screened volunteers, 100% required at least two attempts and 55% required three attempts to correctly answer all questions on the questionnaire. Volunteers completed two written consent forms: one to participate in the study and one to allow the long-term storage of samples taken during the course of the study.

To meet inclusion criteria, all volunteers were required to provide evidence of completion of secondary education (typically completed at age 16-18 years). It was anticipated that participants would have some understanding of the concept of research and might raise different questions and concerns to participants in studies conducted at field sites. It was emphasised that in contrast to many studies, this study would involve making healthy individuals unwell (for a short while). Key concerns volunteers were anticipated to raise were similar to those discussed in screening appointments, namely: concern that the trial was ‘high risk’, the volume of blood taken and the need for an extended in-patient stay (Table 2).

**Table 2:** **Questions raised by volunteers at screening appointments**

**Question category**	**Number of questions asked**	**Percentage (%)**
	**n = 167**	
Effects of malaria infection and treatment: short and long-term	27	16%
Details of in-patient stay and travel out of Nairobi	20	12%
Availability of test results	19	11%
Blood sampling	13	8%
Further explanation of study logistics	13	8%
Parasite strain used in CHMI	13	8%
Prior studies of PfSPZ Challenge	10	6%
Rationale for allocation to groups	9	5%
Malaria treatment	7	4%
Study start date	7	4%
Compensation	6	4%
Post CHMI follow-up	5	3%
Purpose of the research	4	2%
Possibility of failure of infection	3	2%
Criteria for malaria treatment	3	2%
Failure to cure malaria infection	2	1%
Injection sites of PfSPZ Challenge	2	1%
Proof of participation in the study	2	1%
Resistance of PfSPZ Challenge to anti-Malarial drugs	2	1%

#### Compensation for study participation

Volunteers were offered financial compensation for transport expenses and time away from potential income generation or actual work in order to participate in the study. Based on local wages and comparison with the Tanzanian CHMI study initiated in March 2012 [12], this was $50 (Kshs 4,000) for each overnight stay, $12 (Kshs 1,000) for each scheduled clinic visit, and $6 (Kshs 500) for each return journey to clinic. The ERC and OXTREC agreed that these amounts would neither unduly coerce potential participants nor set a difficult precedent for other research conducted within the programme. The availability of financial compensation for enrolled volunteers but not the amount was disclosed at the community sensitization meetings. Only volunteers receiving the VIS at later information meetings were aware of the amount paid as compensation for participation in the study.

#### Conclusion

The informed consent questionnaire was a valuable tool to identify areas where volunteers’ understanding was lacking and facilitate focused discussion with the study investigators. However, the fact all volunteers had to take the questionnaire at least two times suggests volunteers had difficulty understanding the complex study. To rectify this in the future, the length of the information meetings will be extended to allow even more detailed discussion of the study details. The wide range and sophistication of questions asked both at screening and at the community sensitization meetings was a reassuring indication that individuals were engaging with the study. The financial compensation offered to participants did not appear to unduly influence volunteers’ decisions to participate in the study as supported by the fact of the 243 individuals receiving the VIS only 146 attended for screening.

### Recruitment and target population

The occupations of screened volunteers are shown in Additional file 1: Table S2. Despite only promoting the study in medical schools, only 54% of screened volunteers were students, suggesting dissemination of news of the study beyond the planned advertising targets.

**Figure 2:**
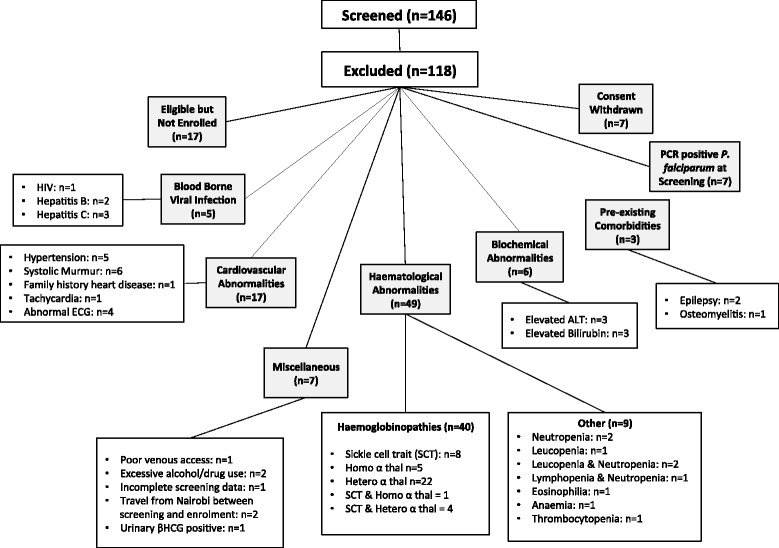
Primary reasons for exclusion of volunteers. Of some individuals met multiple exclusion criteria. This figure illustrates the primary reason for exclusion for each volunteer. ‘HIV’ = human immunodeficiency virus; ‘ECG’ = electrocardiogram; ‘PCR’ = polymerase chain reaction for *P. falciparum*; ‘ALT’ = Alanine transaminase; ‘Homo α thal’ = Homozygous α thalassemia; ‘Hetero α thal’ = Heterozygous α thalassemia; βHCG = β Human Chorionic Gonadotropin.

As the first CHMI study undertaken in Kenya, the exclusion criteria were extensive (Additional file 1: Table S1) and only 45/146 (31%) of the screened volunteers were eligible to participate in the study (Figure 2). Key factors were the high degree of previously undiagnosed co-morbidities and haemoglobinopathies.

#### Haemoglobinopathies

Given the known, significant protective effect of sickle cell trait and both heterozygous and homozygous α-thalassaemia against severe malaria [20,21], volunteers with these conditions were screened and excluded from the study. This did however markedly reduce the proportion of eligible volunteers. As these haemoglobinopathies only confer mild or no protection against mild malaria and asymptomatic parasitaemia [20], future CHMI studies could consider including such volunteers. Indeed, in the recent Tanzanian CHMI study using PfSPZ Challenge, a considerable number of enrolled volunteers were heterozygous for α-thalassaemia and no evidence of an effect on parasite growth dynamics (PGD) was seen post CHMI [12]. An added advantage of including volunteers with these haemoglobinopathies in future CHMI studies is the ability to assess the efficacy of novel vaccines and drugs in participants more representative of the target population at large.

### Classifying prior exposure to *Plasmodium falciparum*

One of the aims of the study was to assess PGD in volunteers with varying degrees of prior exposure, and therefore presumed naturally acquired immunity (NAI) to *P. falciparum* [22]. Although there is little or no natural transmission of malaria in Nairobi [23], the study team were confident of the ability to recruit volunteers with varying degrees of prior exposure to *P. falciparum* given the highly migratory nature of the population of Nairobi.

Volunteers were to be divided into two main cohorts: individuals with evidence of marked prior exposure (i.e., semi-immune; Groups 2, 4 and 6) and individuals with no evidence of prior exposure to *P. falciparum* (Groups 1, 3 and 5) (Figure 1). Given the lack of a known assay to reliably assess NAI [22], serological and historical geographical data were to be used as criteria for grading prior exposure to *P. falciparum,* as a surrogate measure of NAI (Table 3). Data were collected at screening regarding volunteers’ place of birth, location of schooling and higher education and time spent in Nairobi directly prior to screening. Serologically, two key antigens were chosen on the basis of their published, positive association with prior exposure to *P. falciparum:* total schizont antigens and merozoite surface protein 2 (MSP2) [24-28]. Absolute ELISA OD results to these two antigens for screening samples were compared to negative controls (non-exposed UK sera; n = 30) and the cut-off for seropositivity determined as the mean absolute ELISA OD plus three standard deviations of the negative controls (0.23 and 0.07 for reactivity against schizont extract and MSP2, respectively).

**Table 3:** **Initial criteria for group allocation according to degree of prior exposure to** ***Plasmodium falciparum***

**Groups**	**Malaria exposure**	Anti-Pf serology*	**History of malaria infection**	**Time spent in endemic region**
1, 3, 5	None	All negative	None	Lived whole life in Nairobi or similar area where *P. falciparum* is not endemic.
2, 4, 6	Maximal	High positive	+/-	Vast majority of life spent in western Kenya in area of high *P. falciparum* transmission. Immediately prior to CHMI, should not have spent more than 6 months out of endemic region.

*Anti-Pf serology = Anti-schizont and Anti-MSP2 antibodies.

Following collection of data from screening, it became clear the initial criteria for classifying volunteers were unworkable. Firstly, in the target, educated population, most had attended boarding schools and higher education institutions at multiple locations, considerable distances from their place of birth, making it extremely difficult to quantify time spent in malaria-endemic regions (Additional file 1: Tables S3 and S4). Secondly, using CDC data on malaria endemicity (“*P. falciparum*… present in all areas (including game parks) (of Kenya) at altitudes below 2,500 m (8,202ft) with none in the highly urbanized, central part of the city of Nairobi”) [23], nearly all volunteers had spent significant time in a malaria-endemic region (Additional file 1: Tables S3 and S4). Although information on volunteers’ reported history of episodes of blood smear-confirmed malaria infections was collected, it was impossible to validate these reports, since many individuals had taken anti-malarial medications for presumed malaria infection unconfirmed by blood smear and it was difficult to access medical records retrospectively.

On review, it was felt serological data were the most objective measure to assess prior exposure to *P. falciparum*. Given that anti-schizont OD results reflect reactogenicity to multiple antigens in parallel, it was decided to use this assay as the primary serological endpoint. Individuals were, therefore, redefined into those with evidence of minimal prior exposure (MinExp: Groups 1, 3 and 5) and those with definite evidence of prior exposure (DefExp: Groups 2, 4 and 6) on the basis of serological data alone (Table 4). In the final enrolled volunteers, it was ensured that the geographical history was consistent with the serological data (for example, that volunteers reporting lifelong residence in Nairobi were not in the DefExp group).

**Table 4:** **Final criteria for group allocation according to degree of prior exposure to** ***Plasmodium falciparum***

**Groups**	**Malaria exposure**	**Primary criterion:**	**Secondary criterion:**
		Anti-Pf serology*	**Time spent in endemic region**
**1, 3, 5**	Minimal	All negative	Lived majority of life in Nairobi or area where *P. falciparum* is not endemic.
**2, 4, 6**	Definite	Positive anti-schizont +/- positive anti-MSP2	Considerable time spent in areas of *P. falciparum* transmission.

*Anti-Pf serology = Anti-schizont and Anti-MSP2 antibodies.

**Figure 3:**
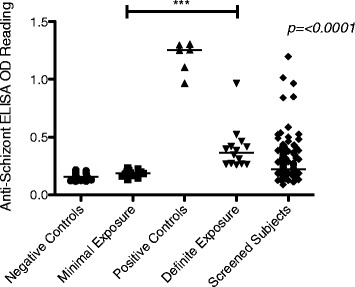
Anti-schizont antibody ELISA absolute OD readings measured at screening. Serum diluted 1:1,000. Negative controls = OD readings from UK malaria-naïve adults (n = 30). Positive controls = OD readings from hyperimmune Kenyan adults living in malaria-endemic regions (n = 6). Minimal exposure = subjects enrolled in Groups 1, 3 and 5 (n = 14). Definite exposure = subjects enrolled in Groups 2, 4 and 6 (n = 14). Screened subjects = all volunteers that had blood drawn at screening (n = 145). Median values represented by lines through each dataset.

On analysis of data, only five screened individuals had anti-schizont OD readings of the same order as ‘hyperimmune’ controls (Figure 3) and due to a high degree of previously undiagnosed co-morbidities in these five individuals, only one of these volunteers was subsequently eligible to participate in the study. In practice therefore, the eligible volunteer pool was made up of subjects with varying but probably nil to mild/moderate prior exposure (and therefore NAI) to *P. falciparum*.

### Asymptomatic parasitaemia at screening

Whilst malaria transmission is minimal in Nairobi [23], it was important to ensure volunteers were not parasitaemic with naturally acquired *P. falciparum* prior to administration of PfSPZ Challenge. Administration of a course of curative anti-malarials to all enrolled volunteers prior to CHMI was considered, however, there were concerns that even a medication with a reported short half-life could impact on PfSPZ Challenge infectivity or PGD [29]. Instead, all volunteers qPCR-positive for *P. falciparum* at screening were excluded (Additional file 1: Table S1). Seven volunteers (5%) were excluded using this criterion (Figure 2). All were asymptomatic: five with a ‘low positive’ unquantifiable result (below the lower limit of quantification for the assay: 20 parasites/mL) and two with quantifiable results of 55 and 669 parasites/mL. These seven volunteers were treated with a course of anti-malarial therapy according to national guidelines and excluded from the study. Whilst it is unclear when these individuals were infected with malaria, they may have been semi-immune volunteers capable of controlling parasitaemia; indeed 4/7 (57%) met the criteria for DefExp. Given the difficulty recruiting such individuals in Nairobi, it was an unfortunate effect of the study design that it was necessary to exclude such volunteers.

#### Conclusion

Given only 54% of screened volunteers were students, it was felt that there was no clear advantage to exclusively targeting medical students and future studies would appeal to students of all disciplines.

Exclusion of volunteers heterozygous for α-thalassaemia markedly limited recruitment and future CHMI studies should consider inclusion of such volunteers. Grouping volunteers according to prior exposure to malaria, and therefore NAI was extremely difficult given the lack of a validated assay and recognized methods to grade malaria exposure on the basis of geographical data. This was compounded by the highly migratory nature of the screened population and high degree of undiagnosed co-morbidities. If future Kenyan CHMI studies seek to enrol ‘hyperimmune’ adults, it will be important to either screen a considerably larger sample of individuals, do more targeted screening or move the study site to an endemic region. Whilst prescreening of volunteers to create a database of appropriate individuals in advance of a CHMI study could be useful logistically, given the unknown dynamics of the loss of NAI, such volunteers would require rescreening ahead of enrolment to ensure accurate, timely data immediately prior to CHMI.

### Study design

#### Dose of PfSPZ challenge

Limited evidence existed at the time of study design to guide the choice of dose and route of administration of PfSPZ Challenge for this study. The dose needed to be large enough to ensure successful infection of all volunteers, but not so large as to overwhelm and prevent the ability of any NAI to control blood-stage growth *in vivo*. Published clinical data at the time of the study design showed IM administration of PfSPZ Challenge to be the only route proven to infect 100% of volunteers [11]. However, the maximum dose used in this study (25,000 PfSPZ) resulted in a notably lower liver-to-blood-inoculum (LBI) than that seen in traditional mosquito-bite CHMI studies [11,18]. In order to ensure infection and detectable parasitaemia post-CHMI, a higher dose of 125,000 PfSPZ IM was chosen for the main study cohort (n = 20). Since this would be the largest dose of PfSPZ Challenge administered IM to humans at the time [11], two additional, small, lead-in cohorts receiving lower doses of 25,000 (n = 4) and 75,000 (n = 4) PfSPZ IM were included in the study design.

Reassuringly, a similar safety profile to that reported in malaria-naïve subjects who underwent CHMI was seen, with the exception that Kenyan participants experienced AEs of a notably longer duration than malaria-naïve volunteers, the reason for which is unclear [13].

#### Schedule of enrolment

In order to minimize vials of PfSPZ Challenge used and ensure standardization with other PFSPZ Challenge studies, staff from Sanaria Inc USA prepared and dispensed syringes of PfSPZ Challenge, which were required to be administered within 30 minutes of thawing of PfSPZ Challenge. For logistical reasons it was decided all volunteers would undergo CHMI in the same week. Given a safety review by the Safety Monitoring Committee (SMC) was required prior to each dose escalation of PfSPZ Challenge, safety data had to be compiled in real-time and SMC members briefed in advance to allow a strict schedule of reviews prior to approval of each dose escalation to be met (Additional file 1: Table S5). The fact that all volunteers were enrolled in one week meant that the phase of in-patient follow-up lasted just 28 days and so intensive nursing and laboratory support was only required for a month.

#### In-patient setting

Depending on the setting, management of subjects undergoing CHMI is done on an patient or out-patient basis [14]. Given the need to prioritize volunteer safety in this pilot study, the fact that considerable traffic congestion in Nairobi could seriously impede volunteers’ ability to attend the medical centre in a timely fashion and the lack of ease of access to public health care, it was decided that volunteers would be managed as in-patients from the day before injection of PfSPZ Challenge until completion of anti-malarial therapy. The need for in-patient care was emphasized to participants at screening and again prior to enrolment. Clear protocols were established and explained to both staff and participants regarding the actions to be taken if a participant were to go missing [30]. Participants were encouraged to voice any concerns they had about their living conditions, treatment or change in personal circumstances early so that issues could be addressed quickly and anti-malarial treatment initiated in a timely fashion if a volunteer wished to withdraw from the study and leave the in-patient clinic.

#### Duration of follow-up post CHMI

**Figure 4:**
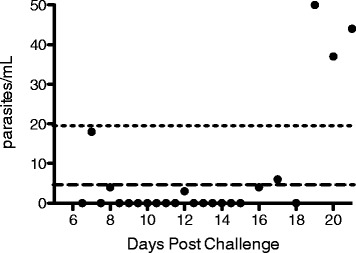
qPCR results post-challenge for Volunteer 110, Group 2. Long dashed line = lower limit of detection (i.e., a probability of > 50% of ≥ 1 positive result among three replicate PCR reactions) for qPCR assay (5 parasites/mL). Short dashed line = lower limit of quantification (defined as %CV < 20%) for qPCR assay (20 parasites/mL).

As the first pilot study in Kenya, a follow-up schedule similar to European centres was adopted, with all undiagnosed participants treated at C+21 [14]. One participant (110) who had an anti-schizont ELISA OD at screening similar to hyperimmune individuals was qPCR-positive but blood film-negative (and therefore undiagnosed) on C+21 (Figure 4). It would be extremely interesting to examine the PGD of this, and other hyperimmune individuals if a longer window of follow-up prior to treatment of undiagnosed participants were adopted. One benefit could be the ability to track the development of gametocytaemia. In this study, no qPCR specific for gametocytes was performed, and so it is possible that the rise in qPCR seen in participant 110 from C+19 onwards reflected gametocytaemia rather than loss of control of the blood stage of infection. In future studies, if qPCR specific for gametocytes were performed and a phenotype of individuals identified that were able to control blood-stage infection, remain asymptomatic but develop gametocytes, this could allow for the development of a much needed clinical model to assess the efficacy of novel transmission-blocking vaccines [31].

In this study, the risk of spread of the challenge strain of *P. falciparum* (NF54) into the surrounding area was thought to be minimal due to treatment of participants prior to the typical development of gametocytes and the lack of an appropriate mosquito vector in Nairobi. However, if the follow-up period prior to treatment were extended in future studies, particularly in sites with local transmission of *P. falciparum*, careful thought should be given to the potential for natural transmission of the challenge parasite to local areas.

#### Sequencing of parasite at diagnosis

In contrast to CHMI studies conducted at northern centres, MSP2 genotyping [32] of the parasites collected at diagnosis was performed to ensure all infections were clonal (NF54) and resulting from PfSPZ Challenge rather than natural transmission.

##### Conclusion

In this study, the need to enrol all volunteers within one week and confirm this date in advance in order to coordinate with staff from Sanaria Inc did limit the time available for screening. If local staff were trained to thaw and dilute PfSPZ Challenge and load syringes, then enrolment could be spread over a longer period, which would be especially useful if there is a difficulty recruiting appropriate participants. This should be possible as more of these studies are performed in centres in malaria-endemic countries.

All participants in this study were successfully infected, however LBIs were notably lower (4,415-106,484 parasites) [13] than those typically seen in mosquito-bite CHMI studies (240,000-2,835,000 parasites depending on the CHMI centre) [18]. Recent trials using PfSPZ Challenge administered intravenously (IV) by direct venous inoculation (DVI) in 0.5 mL suggest this route of administration can reliably ensure successful infection in CHMI trials with lower doses of sporozoites than those required when administered by other routes [33], and indeed 3,200 PfSPZ administered by DVI is now the preferred dose and route being used currently to assess a number of novel vaccines (SLH, pers comm). However, until a vaccine’s efficacy is assessed both in a PfSPZ Challenge CHMI study and in a field study, it will be unknown if the PfSPZ Challenge model accurately predicts vaccine efficacy in a field setting.

Future CHMI studies aiming to examine the dynamics of NAI or the efficacy of blood stage or transmission-blocking vaccines should consider treating undiagnosed volunteers later than C+21. Performing qPCR in real time to inform treatment decisions and measuring gametocytaemia using specific qPCR could also increase the applications of the CHMI model in Africa [7]. All CHMI sites in areas of *P. falciparum* transmission should routinely perform genotyping on parasites collected at diagnosis to ensure superadded naturally acquired infection has not occurred.

Using qPCR in addition to microscopy is important to distinguish uninfected smear-negative volunteers from those smear negative volunteers with a parasitaemia below the threshold of detection by microscopy.

### Clinical and laboratory resources

Given the complex and unique nature of CHMI studies, in particular the need for results to be available in real time to guide clinical decision making, it was critical that both clinical and laboratory staff received detailed training in advance of the study. This involved the creation of 22 clinical and 29 laboratory study-specific standard operating procedures (SOPs) (Additional file 1: Tables S6 and S7), weeks of dedicated training sessions and ‘dry runs’ of the preparation and administration of PfSPZ Challenge. In order to adhere to internationally agreed standards [14] and achieve consistency with northern CHMI centres, significant clinical and laboratory training and support were provided by both national and international collaborators. Since blood film positivity was the primary criteria for the start of treatment, microscopy was a critical area of training. All microscopists passed a two-week refresher-training course at the KEMRI-affiliated Malaria Diagnostics Centre of Excellence, Kisumu, Kenya approximately three months prior to working on study samples. Blood smears were prepared and interpreted according to a modified version of the “Consensus SOP for Malaria Microscopy in the Context of Clinical Challenge Trials” [13,14]. It was particularly important in this study to emphasize to the microscopists, who were used to working in a clinical, field setting, the importance of avoiding false positive results.

#### Conclusion

Support from established CHMI centres and detailed advanced training was essential for the first Kenyan CHMI study to take place safely and efficiently.

### Feedback

On discharge, all participants were asked to complete an exit questionnaire providing feedback on their experience participating in the study. Nineteen of 28 (68%) participants returned the anonymized questionnaire. Results were positive (Additional file 1: Table S8) with participants reporting good understanding of the study and discharge procedures.

A written final report will be submitted to the ethical and regulatory bodies that approved the study and the results of the study presented to university managers, local students and interested study participants when all analyses of study related data is completed.

#### Conclusion

Whilst this first pilot study in Kenya, it is the start of an iterative process, which will be informed by these experiences and feedback from volunteers, ethics and regulatory bodies and collaborators. In particular, the VIS will be revised to include more detailed information regarding the issues raised by volunteers. It will also be crucial to feedback findings of the study to the key stakeholders in order to help maintain a working relationship for future CHMI studies.

## Conclusion

There is a current need to increase the international capacity for efficacy testing of candidate malaria vaccines and allow earlier assessment of novel vaccines and drugs in the target African populations [7]. The establishment of the CHMI model in African centres is one key strategy that could help address these aims [8]. This study has shown that sporozoite CHMI studies using PfSPZ Challenge can be safely performed in Kenya in individuals with varying degrees of prior exposure to malaria [13].

However, performing CHMI studies in an African setting presents unique but surmountable challenges. As the first pilot study performed in Kenya, there were many learning points, most unique to the African setting and this paper provides key feedback to aid other African sites wishing to establish the CHMI model. In addition, there remain key scientific questions about PfSPZ Challenge, such as whether protection as assessed by DVI is comparable to or correlated with protection assessed by mosquito bite CHMI or natural transmission, both of which lead to deposition of a portion of the parasites in the skin. Given the CHMI model has been shown to be safe and feasible in Kenya, there is hope the international CHMI and malaria vaccine community will continue to support the Kenyan site and others to allow on-going collaborative frameworks to help address these questions, and to accelerate the development of candidate malaria vaccines in malaria-endemic populations.

## Additional file

Additional file 1:
**Supplementary information.**

## Abbreviations

C-1: Day before CHMI
C + 21: 21 days post CHMI
CDC: US Centers for Disease Control and Prevention
CHMI: Controlled human malaria infection
DefExp: Definite prior exposure to *P. falciparum*
DVI: Direct venous inoculation
ELISA: Enzyme linked immunosorbent assay
ERC: KEMRI Ethics Review Committee
FDA: US Food and Drug Administration
HIV: Human Immunodeficiency Virus
IV: Intravenously or intravenous
KEMRI: Kenya Medical Research Institute
LBI: Liver-to-blood inoculum
MinExp: Minimal prior exposure to *P. falciparum*
MSP2: Merozoite surface protein 2
NAI: Naturally acquired immunity
OD: Optical density
OXTREC: Oxford Tropical Research Ethics Committee
PfSPZ Challenge: Aseptic, purified, cryopreserved, infectious *P. falciparum* sporozoites
PfSPZ: *Plasmodium falciparum* sporozoites
PGD: Parasite growth dynamics
qPCR: quantitative polymerase chain reaction
SMC: Safety Monitoring Committee
SPZ: Sporozoites
WHO: World Health Organization
VIS: Volunteer information sheet
