# Supplementary material for: Lessons learnt from the first controlled human malaria infection study conducted in Nairobi, Kenya
Source: Malar J. 2015 Apr 28;14:182. doi: 10.1186/s12936-015-0671-x (PMC4416324; doi:10.1186/s12936-015-0671-x)
Supplement: Additional file 1: — Supplementary information. [file 12936_2015_671_MOESM1_ESM.docx]

**Supplementary Information**

PARTICIPANT INFORMATION AND CONSENT FORM

**A study to examine controlled human malaria infection, administered by needle and syringe in African adults**

**Protocol Version: 7.0 3^rd^ April 2013**

**PART 1: INFORMATION SHEET**

**PRINCIPAL INVESTIGATORS:** Bernhards Ogutu and Kevin Marsh

**INVESTIGATORS AND INSTITUTIONS**

***KEMRI:*** Susanne Sheehy, Elizabeth Juma, Faith Osier, Patricia Njuguna, Catherine Molyneux, Andrew Cole, Caroline Ogwang

***The Centre for Tropical and Travel Medicine, Nairobi:*** Charles Chunge.

***University of Oxford:*** Adrian Hill, Alison Lawrie.

***Sanaria:*** Stephen Hoffman, Peter Billingsley, Kim Lee Sim, Eric James

**What is KEMRI?**

KEMRI is a government organization under the Ministry of Health, which carries out medical research. Research is different from normal treatment because research aims to find better ways of preventing and treating illness in the future for everybody’s benefit. One of the projects in KEMRI is to contribute to increased understanding of the body’s immune response to malaria. We are looking for volunteers to receive malaria infection under carefully controlled conditions so that we can examine the immune response to malaria. It is hoped the information gained from this study will help development of a malaria vaccine.

## What is this research about?

Malaria is a common and serious disease in young children that results in many childhood deaths. Although malaria is becoming less frequent in some parts of Africa as bed nets and anti-malarial drugs become more available, vaccines are still needed to prevent malaria as it is still a major problem.

An important part of malaria research is malaria challenge studies. This is when healthy volunteers are infected with malaria (‘challenged’) under carefully controlled conditions. Challenge studies have been conducted for 15 years in America, Europe and Australia but have not yet been conducted in Africa; however a challenge study is planned in Tanzania in mid-2012.

In this study we plan to ‘challenge’ healthy African adults with malaria and then closely examine the immune response. You will be given malaria by injection of malaria parasites into the muscle in your upper arms. These parasites have been developed by American researchers and have been given to volunteers in Holland and the UK. Following injection you will stay in our clinical research facility in Nairobi full time until you are treated for malaria. We hope that the information we get from this study will help malaria vaccine development.

We hope to recruit a minimum of 28 volunteers who may all develop malaria. All volunteers will be injected with malaria parasites. All participants will receive two injections at the same time. The number of parasites given to volunteers will vary. All volunteers will be followed up closely to observe if and when they develop malaria. When they develop malaria they will be treated with antimalarial drug that is known to be effective for treatment.

## Do I have to take part?

No. It is up to you to decide whether or not to take part. If you do decide to take part, you will be asked to complete a questionnaire assessing your understanding of the study in order for us to be confident that you fully understand what taking part will involve. You need to answer all questions correctly in order to take part in the study. If you don’t answer all the questions correctly the first time, you will be able to complete the questionnaire again after discussion with the Investigator. You will then be asked to sign a consent form. You are free to withdraw at any time without giving a reason, but you may be asked to return to the clinic for follow up for safety reasons.

**Am I eligible to be involved in the trial?**

In order to be involved in the study you must be:

- A healthy adult aged between 18 to 40 years.
- Have completed a minimum of 4 completed years (Form 4) of secondary education.
- Able and willing (in the investigators’ opinion) to comply with all study requirements.
- Willing to stay in KEMRI Centre for Clinical Research (CCR), Nairobi for a part of the study (from day of administration of malaria parasites until completion of a course anti-malaria therapy – this would be between 9 and 23 days).

### You cannot participate in this study if:

- You are intending to leave Nairobi between screening appointment and completion of malaria challenge.
- You have used antibiotics which could treat malaria in the 30 days prior to involvement in the study.
- You have previously received an investigational malaria vaccine.
- You have had immunoglobulins and/or any blood products in the three months preceding your involvement this trial.
- You have problems with your immune system.
- You have a red blood cell abnormality that may affect study outcome.
- You are pregnant, breast feeding or intend to become pregnant during the study.
- You have allergic disease or reactions likely to be exacerbated by malaria infection.
- You have a history of cancer.
- You have a history of a serious psychiatric condition that may affect participation in the study.
- You have any other serious chronic illnesses.
- You have injected drugs at any time in the last 5 years.
- You have hepatitis B, hepatitis C or infection.
- You have a history of heart disease.
- Close family members have developed heart disease when aged younger than 50 years.

Mild conditions, such as childhood asthma which is well controlled would not automatically exclude you from participating. If you are unclear whether you are eligible to be involved in the study you can contact the study team who will be able to advise you.

**What will it involve for me?**

If you agree to take part in this study, the following will happen:

1. **Screening Appointment**: A member of the clinical team will discuss the trial with you and answer any questions you have. If you decide to participate, you will be asked to complete a questionnaire to assess your understanding of the study and to sign a consent form. The clinician will then check your general health by asking questions and examining you. After this blood samples will be taken for testing. 18mls (about 1 tablespoon) of blood will be taken from your arm for this purpose. A test of your urine will also be performed and this will include a pregnancy test for women. If we find you have any health problems or abnormal blood tests, we will explain this to you and refer you for any further tests or treatment you need at the most appropriate health facility. We will not ask you to continue to participating in this study.

**HIV testing**

One of these blood tests will be an HIV test. A trained counsellor will explain the HIV test to you before it is done, and discuss the results with you afterwards. All results will be kept confidential. You will be given your results in private. If you are HIV positive, you will be referred to a local health facility where you will receive standard medical care according to the ministry of health guidelines.

If you are found to be fit to receive the injection of malaria parasites the study will involve the following activities:

1. **Repeat Healthy Check Day before Injection of Malaria Parasites:** We will ask you to return to clinic for a review, blood test and, for female volunteers, a urinary pregnancy test a day or two before the challenge to ensure you are completely healthy and can enter the malaria trial. We will also collect a 68mls (4.5 tablespoons) blood sample at this time.
2. **Injection of Malaria Parasites & In-patient Stay at Study Clinic:** You will be given two injections of malaria parasites, one in each of your upper arms. You will either receive a low, medium or high dose of parasites. **After this injection you will stay in the clinic at KEMRI Centre for Clinical Research, Nairobi, full time until you have completed malaria treatment. You will be expected not to leave the clinic during this time and to sleep in the clinic.**
3. **Health Checks and Blood Tests Following Injection of Malaria Parasites:** After injection of the parasites, you will be reviewed at least twice a day by the study staff. But if you feel unwell then you will be reviewed more often. A nurse or doctor will be available 24 hours a day in the clinic so there will always be someone to look after you. Days 1-5 following injection of malaria parasites no blood samples will be taken. On days 6-23 following injection of malaria parasites, a blood test will be taken once or twice day. The total amount of blood taken each time will vary but will be between 3ml and 31mls (a few drops and 2 tablespoons).

As soon as you are diagnosed with malaria you will be started on a 2 day course of anti-malaria tablets. We will then do a blood test 24 and 48 hours after starting anti-malaria tablets to make sure the malaria infection is being treated.

If you become unwell with malaria then you may be admitted to a Nairobi Hospital as a precaution until you have recovered, but it is very unlikely that this will be necessary. The hospitals you may be admitted to include; Nairobi hospital, Aga Khan University Hospital and Mater Hospital. You should be diagnosed with malaria between 9-14 days after injection of malaria parasites. In the unlikely event that we do not diagnose you by day 21 after injection of parasites then we will give you a treatment course of antimalarial treatment anyway so that any parasites that we have not detected will be killed.

As long as you are well and there are no malaria parasites in these blood tests you can leave the clinic 48 hours after starting malaria treatment. So the longest time you could spend in the clinic is approximately **23 days**.

1. **Follow-up visits:** We will ask you to return to clinic for a health check 35 days and 90 days after injection of malaria parasites. We will take a blood test at these visits of 25 or 35 mls (approximately 2 tablespoons). In case you feel unwell while at home you should contact the study our clinic or come for review.

**What Amount of Blood Will Be Taken During the Study?**

The total amount of blood taken during the study will be 433 mls (29 tablespoons) over approximately 3 months. These blood tests are to assess your immune response to malaria. The amount taken at each visit will vary between 3ml and 68 mls (a few drops and 4.5 tablespoons). This amount of should not cause any problems in healthy people. We will give you a copy of your blood tests if you request them.

The amount of blood taken at each visit is outlined in the following table:

| Schedule | Screening Visit | Day Before Injection | Days 6-14 post injection | Days 15-23 post injection | Day 35 post injection | Day 90 post injection |
| --- | --- | --- | --- | --- | --- | --- |
| Blood volume per visit (mL) | 18 | 68 | 3-28 | 3-36 | 38 | 25 |
| Blood volume per visit (tablespoons) | 1 | 4.5 | 1/5 - 2 | 1/5 - 2 | 2.5 | 2 |
| Cummulative blood volume (mL) | 18 | 86 | 234 | 370 | 408 | 433 |

**Considerations before Taking Part in the Study:**

1. **Medications:** You should not take any drugs other than vitamin pills, contraceptive pills or those medications assessed as appropriately safe during a malaria challenge by the doctor at screening. This also applies for drugs bought over the counter. Of course, your health and well-being is much more important than the conduct of this study and if at any time you need any medication then you should take it. However, it is very important that you **let us know before you start on any treatment**. For example, any antibiotics that you take within 4 weeks of the planned challenge day may affect the malaria parasite and mean that we cannot include you in the study.
2. **Travel Outside Nairobi:** We will test your blood for malaria parasites at your screening visit as there need to be no malaria parasites in your blood for you to be in the study. It is very important that you do not become infected with malaria between your screening visit and the day of injection of malaria parasites. While it is not possible to catch malaria in Nairobi, it is possible to catch malaria in many other parts of Kenya. We therefore ask you to you not to leave Nairobi between your screening visit and day of injection with malaria parasites.
3. **Pregnancy:** Malaria infection can be particularly dangerous during pregnancy to both the mother and the baby. Women are therefore asked to use an effective method of contraception such as pills or condoms during study period to avoid pregnancy. If you are not already using any contraception and require it, you will be referred to the nearest family planning clinic to obtain them. A pregnancy test will be carried out at screening, before the injection with malaria parasites and again before anti-malarial treatment is started.
4. **Blood Donation:** You should not donate blood whilst you are taking part in this study.

**Are there any risks or disadvantages to me for taking part in the study?**

Our priority for every participant is their well-being. However there are some potential risks from taking part in the study;

1. **Blood Taking:** There may be some pain and bruising associated with blood drawing which will resolve after a few days. There is a small risk of infection. This risk is minimized by use of prepackaged sterile equipment and trained staff.
2. **Injection of Malaria Parasites:** You may experience some injection site pain, redness or warmth where the parasites are injected however this is most likely to be mild. With any injection there is a risk of a severe allergic side effect. These include skin swelling, shortness of breath and light-headedness or fainting. Medical equipment necessary to treat serious reactions will be available in clinic. You will be closely monitored by a study doctor who will deal with any injection related illness should they occur.
3. **Malaria Infection:** The risks of taking part in this study are very low providing that you stay in the clinic after injection of malaria parasites so that the study team can look after you. However, if untreated, the malaria infection that we propose to give you could result in death. Worldwide over 1300 people have been deliberately infected with malaria in challenge studies to date and all have made a complete recovery.

We hope to diagnose and treat your malaria infection before the onset of symptoms however it is likely you will develop some symptoms of malaria, such as a flu-like illness, fever, chills, headache, muscle pains, diarrhoea or vomiting. These symptoms may be severe enough that you need to be in bed for a couple of days. We can prescribe pain-killers such as paracetamol and anti-sickness tablets if required. Symptoms can start or persist after treatment has started but usually last no more than 1 to 3 days.

If malaria is not properly treated, possible complications include jaundice, kidney failure, fluid on the lung, low blood sugar and collapse. Seizures, altered consciousness, coma and even death may occur. It is therefore very important you stay in the inpatient clinic once we have given you the injection of malaria parasites and that if you want to leave the study you allow us to treat you first.

For 6 months after the injection of malaria parasites you should contact the study team if you develop any of the symptoms of malaria as detailed above.

The malaria parasites used in this study are known to be very sensitive to the drugs that will be used in the treatment of infection and possible infection. The drugs are all recommended for the treatment of malaria. We therefore do not expect any treatment failures as this has not happened in previous studies.

1. **Treatment of Malaria:** The drug you will be treated with is called Malarone®. Malarone® is a combination drug consisting of 250mg atovaquone and 100mg proguanil hydrochloride per tablet. A treatment course of Malarone® consists of 3 doses of 4 tablets. The first 4 tablets will be given when you are diagnosed with malaria followed by doses 24 and 48 hours later (4 tablets each time). Malarone is a recommended treatment for malaria. We will need to watch you take each of these doses and will continue taking blood to look for parasites until 2 blood tests are negative for malaria parasites. In case you may not be cured with Malarone you will be treated with artemether/lumefantrine which is a recommended treatment for malaria in Kenya.

Like all medicines, Malarone® can cause side effects, although not everybody gets them. Most side effects reported have been mild and have not lasted very long. Very common side effects include headache, nausea and vomiting, stomach pain and diarrhoea. Common side effects include dizziness, insomnia, strange dreams, depression, loss of appetite, fever and rash which may be itchy and cough. Uncommon side effects include anxiety, palpitations, swelling and redness of the mouth and hair loss. Severe allergic reactions have occurred in a small number of people, but their exact frequency is unknown. Signs of severe allergic reactions include rash and itching, sudden wheezing, tightness of the chest or throat, or difficulty breathing, swollen eyelids, face, lips, tongue or other part of the body.

Allergy to the components of Malarone® may prevent you being treated with Malarone®. Please let us know if you think this might apply to you. If need to stop taking Malarone® during the study then you will be treated with another effective medication called CoArtem®.

Paracetamol may be given to you to reduce fever, muscle and joint pain, back ache and headache. If you get any side effects from the malaria medicine such as itching, nausea or vomiting, you will be given another medicine called cyclizine to help relieve those symptoms.

1. **Other Potential Complications:** Two volunteers in the Netherlands a man in 2013 and a lady in 2008 taking part in a malaria vaccine studies were given malaria sporozoites by mosquito bite. They developed malaria and while on treatment for malaria, they developed chest pain. They were both treated in hospital and recovered fully. The man was diagnosed with a condition called “myocarditis” which is an inflammation of heart muscle. The woman was diagnosed with a heart problem, possibly caused by myocarditis or decreased blood flow to the heart muscle. We do not know whether their illness was related to malaria infection (given by mosquito bite), the vaccine that they received, the medicine used to treat malaria, a viral infection unrelated to the study or something else. It has not been reported in volunteers in other centres. We will perform a heart examination called an electrocardiogram (ECG) at screening and once enrolled, if you develop any chest pain, we will transfer you to hospital for appropriate observations and treatment.
2. **Travel & Time:** This study will involve your time and you may have travel costs. However we will compensate for your time and travel expenses with a payment of;
   1. Ksh 500 for travel costs for each journey to clinic
   2. Ksh 1,000 for each clinic visit
   3. Ksh 4,000 for each overnight stay at clinic.

**Are there any benefits for taking part?**

If you participate in this study, you will receive medical care for any acute ailments from the day of injection of malaria parasites until the completion of the study free of charge. Treatment of chronic illnesses or long term injuries unrelated to the study procedures will not be paid for by the study. If you are found to have such illnesses/injuries, you will be treated under the existing government programs.

By participating in this study, you will be helping with malaria research that may help development of a malaria vaccine that would bring health benefits to future generations of children.

**What will happen if I refuse to participate?**

All participation in research is voluntary. You are free to decide if you want to take part or not. If you do agree to participate, we would like you to understand that you are free to change your mind at any time and withdraw from the research. This will not affect your health care now or in the future. **However, if you decide to withdraw from the study after we have given you an injection of malaria parasites then you must agree to take a course of anti-malaria therapy.**

In the event that you leave the study before its completion, we still encourage you to have the examinations and blood tests to assess safety following injection of malaria parasites. This will involve clinic visits at 35 and 90 days following injection of malaria parasites.

**What happens to my blood samples?**

Your blood samples will be coded with your study identification number and no personal information such as your name or date of birth will be included. This is to ensure that samples can only be linked to the participants by people closely concerned with the research.

Samples will be processed in KEMRI (in Nairobi, Kisumu and Kilifi), in Oxford, UK, in the USA by the biotechnology company Sanaria and University of Maryland and in the Netherlands by the University of Nijmegen. All the institutions that samples will go to are collaborators involved in this study and development of a malaria vaccine. Any remaining samples will be stored at our research laboratories in KEMRI. In the future, new research may be done on these stored samples. Any future research will first be approved by KEMRI Ethics Committee to ensure that participants’ safety and wellbeing are protected.

**Will any genetic tests be done?**

Yes. Some blood will be used to look at DNA in your body that makes your body respond to the malaria infection. These DNA are usually in groups called genes and will test for those that are usually responsible for immune response to malaria. This will help us better understand how the immune responses to infection are produced.

**Who will have access to information about me in this research?**

All our research records are stored securely in locked cabinets and password protected computers. Only the people who are closely concerned with the research will be able to view information from participants, in order to be sure that the study is being run correctly and the health of every participant is protected. These individuals will keep the information confidential.

You will be provided with a copy of your signed informed consent.

**Who has allowed this research to take place?**

This study had been approved by local and international committees including expert committees within KEMRI to make sure the research is conducted properly and that participants’ safety and rights are respected. They have looked carefully at this work and agreed that the research is important, relevant to Kenya and follows nationally and internationally agreed guidelines.

The local and international committees will be informed about any serious side effects that are noticed, and if we receive new information about the malaria parasites during the course of the trial, we will inform you.

A description of this clinical trial will be available on the Pan African Trials Registry [**http://www.pactr.org**](http://www.pactr.org)**.** This Web site will not include information that can identify you. At most, the Web site will include a summary of the results. You can search this Web site at any time.

**What if I have any questions?**

You may ask any of our staff questions at any time. You can also contact those who are responsible for this research:

1. Dr Elizabeth Juma: KEMRI, P.O Box 54840-00200, Nairobi, Kenya Telephone: 0722796494
2. Dr Bernhards Ogutu: KEMRI, P.O Box 2254 – 0202 Nairobi, Nairobi, Kenya. Telephone: 0733966065

**If you want to ask someone independent anything about this research please contact:**

The Secretary - KEMRI/National Ethics Review Committee, P. O. BOX 54840-00200, Nairobi, Tel number: 020 272 2541 Mobile: 0722205901 or 0733400003

This research is supported by The University of Oxford and KEMRI, who will pay for any treatment or compensation in the unlikely event of any injury resulting from this trial.

**A study to examine controlled human malaria infection, administered by needle and syringe in African adults**

# INFORMED CONSENT QUESTIONNAIRE

This questionnaire is designed to test your understanding of the study in order for us to be confident that you fully understand what taking part will involve. Please make sure you have read the information sheet in full and asked the Investigator any questions you may have. You need to answer all questions correctly in order to take part in the study. If you don’t answer all the questions correctly the first time, you will be able to complete the questionnaire again after discussion with the Investigator.

Volunteer Name:.…………………….. Volunteer Trial Number:…..………. Date………………Time.…….

**Please clearly circle one answer for each question;**

**1. By participating in this study you can expect to develop which of the following:**

A. Tuberculosis

B. Malaria

C. Typhoid

**2. The study involves volunteers being given malaria by:**

A. Spider bite

B. Mosquito bite

C. Injection

**3. Is it likely that a single treatment course will be effective to treat malaria in this study?**

A. Yes

B. No

**4. Medical screening for this study will include which of the following?**

A. Laboratory tests (including an HIV test)

B. Physical examination

C. Review of medical history

D. All of the above

E. None of the above

**5. If you wish to withdraw from the study you may:**

A. Withdraw voluntarily at any time provided you complete a course of anti-malarial therapy (if needed)

B. Withdraw from the study only if the investigators say it is ok

C. Never withdraw from the study

**6. Which of the following are true regarding pregnancy and participation in this study?**

A. Pregnant women may participate in this study

B. Women should not get pregnant for 12 months after getting malaria

C. An effective method of birth control is required for women while participating in this study

**7. What are common symptoms associated with malaria infection?**

A. Fever

B. Chills

C. Headache

D. All of the above

E. None of the above

**8. How is malaria diagnosed in the study?**

A. Looking at a sample of blood under a microscope

B. Chest X-ray

C. Having you walk on a treadmill

**9. If you develop malaria, we will:**

A. Treat you immediately with effective medications

B. See how sick you can get without treating you

_____________________________________________________________

Volunteer signature / date

Score: _________

Reviewer signature / date_________________________________________

**Supplementary Table 1: Occupations of Screened Volunteers (Data collected for n=145)**

| **Occupation Category** | **Frequency (n)** | **Percentage (%)** |
| --- | --- | --- |
| Student | 78 | 54% |
| Unemployed | 25 | 17% |
| Business | 6 | 4% |
| Casual Labourer | 4 | 3% |
| None/ Not Specified | 4 | 3% |
| Researcher | 4 | 3% |
| Social Worker | 3 | 2% |
| Acrobat | 1 | 1% |
| Broker | 1 | 1% |
| Community Health Worker | 1 | 1% |
| Contractor | 1 | 1% |
| Counsellor | 1 | 1% |
| Driver | 1 | 1% |
| Engineer | 1 | 1% |
| Footballer | 1 | 1% |
| Housekeeper | 1 | 1% |
| Journalist | 1 | 1% |
| Nurse | 1 | 1% |
| Online Writer | 1 | 1% |
| Plumber | 1 | 1% |
| Sales and Marketing | 1 | 1% |
| Sales and Services | 1 | 1% |
| Secretary | 1 | 1% |
| Self Employed | 1 | 1% |
| Stylist & Beautician | 1 | 1% |
| Teacher | 1 | 1% |
| TV Producer | 1 | 1% |
| Waitress | 1 | 1% |

**Supplementary Table 2: Inclusion and Exclusion Criteria for the Kenyan Controlled Human Malaria Study**

| **Inclusion Criteria** |
| --- |
| - Healthy adults aged 18 to 40 years. - Minimum of 4 completed years of secondary education. - Able and willing, in the Investigator’s opinion, to comply with all study requirements. - Informed consent to undergo CHMI. - Answer all questions on the informed consent questionnaire correctly. - Willingness to take a course of curative anti-malaria medication. - Agreement to stay in an in-patient unit during a part of the study (from day of administration of PfSPZ Challenge until completion of curative course of anti-malarial therapy given either at malaria diagnosis or day 21 post-administration of PfSPZ Challenge). - Use of effective method of contraception for duration of study (women only). |
| **Exclusion Criteria (any of the following)** |
| - PCR positive for *P. falciparum* parasites at screening. - Travel to a malaria endemic region between screening and enrolment. - Use of systemic antibiotics with known antimalarial activity within 30 days of administration of PfSPZ Challenge (e.g. trimethoprim-sulfamethoxazole, doxycycline, tetracycline, clindamycin, erythromycin, fluoroquinolones and azithromycin). - Receipt of an investigational product in the 30 days preceding enrolment, or planned receipt during the study period. - Current participation in another clinical trial or recent participation within 12 weeks of enrolment. - Prior receipt of an investigational malaria vaccine. - Any confirmed or suspected immunosuppressive or immunodeficient state, including HIV infection; asplenia; recurrent, severe infections and chronic (more than 14 days) immunosuppressant medication within the past 6 months (inhaled and topical steroids are allowed). - Use of immunoglobulins or blood products within 3 months prior to enrolment. - Sickle cell trait or heterozygous or homozygous alpha thalassemia. - A history of allergic disease or reactions likely to be exacerbated by malaria infection. - Contraindications to atovaquone/proguanil hydrochloride. - History of cancer (except basal cell carcinoma of the skin and cervical carcinoma *in situ*). - History of serious psychiatric condition that may affect participation in the study. - Any other serious chronic illness requiring hospital specialist supervision. - Women only; pregnancy, intention to become pregnant or breast-feeding during study. - Suspected or known current alcohol abuse. - Suspected or known injecting drug abuse. - Seropositive for hepatitis B surface antigen (HBsAg). - Seropositive for hepatitis C virus (antibodies to HCV) with PCR positive for Hepatitis C. - Positive family history in 1st and 2nd degree relatives < 50 years old for cardiac disease. - Any clinically significant abnormal finding on biochemistry or haematology blood tests, urinalysis or clinical examination.   Any other significant disease, disorder or finding which may significantly increase the risk to the volunteer because of participation in the study, affect the ability of the volunteer to participate in the study or impair interpretation of the study data. |

**Supplementary Table 3: Enrolled Volunteers in Groups 1, 3 and 5 (n=14)**

| **Subject** | **Group** | **Place of Birth (POB)** | **Years in POB** | **Location Childhood** | **Location Primary School** | **Location High School** | **Location Higher Education** | **Location Most Time Spent** | **Location 3 Years Prior to CHMI** | **Years in Nairobi Prior to CHMI** | **Time Spent Malaria Endemic Region** |
| --- | --- | --- | --- | --- | --- | --- | --- | --- | --- | --- | --- |
| 167 | 1 | EMBU | 20 | EMBU | EMBU | EMBU | NAIROBI | EMBU | NAIROBI | 6 | Y |
| 228 | 1 | NAIROBI | 24 | NAIROBI | NAIROBI | NAIROBI | NAIROBI | NAIROBI | NAIROBI | 24 | N |
| 133 | 3 | NAIROBI | 19 | NAIROBI | NAIROBI | NAIROBI | N/A | NAIROBI | NAIROBI | 19 | N |
| 197 | 3 | ELDAMA RAVINE | 15 | ELDAMA RAVINE | ELDAMA RAVINE | MASENO | NAIROBI | ELDAMA RAVINE | NAIROBI | 3 | Y |
| 117 | 5 | NAIROBI | 31 | NAIROBI | NAIROBI | MURANG'A | NAIROBI | NAIROBI | NAIROBI | 31 | Y |
| 118 | 5 | KAKAMEGA | 15 | KAKAMEGA | KAKAMEGA | NAIROBI | NAIROBI | KAKAMEGA | NAIROBI | 4 | Y |
| 127 | 5 | NAIROBI | 24 | NAIROBI | NAIROBI | NAIROBI | NAIROBI | NAIROBI | NAIROBI | 24 | N |
| 132 | 5 | MURANG'A | 17 | MURANG'A | MURANG'A | MURANG'A | NAIROBI | MURANG'A | NAIROBI | 10 | Y |
| 142 | 5 | MURANG'A | 6 | MURANG'A | NAIROBI | KIAMBU | NAIROBI | NAIROBI | NAIROBI | 20 | Y |
| 189 | 5 | NAIROBI | 24 | NAIROBI | NAIROBI | NAIROBI | NAIROBI | NAIROBI | NAIROBI | 24 | N |
| 205 | 5 | NAKURU | 17 | NAKURU | KINARI | NGORIKA | NAIROBI | NAKURU | NAIROBI | 7 | Y |
| 216 | 5 | NAIROBI | 20 | NAIROBI | NAIROBI | KIAMBU | NAIROBI | NAIROBI | NAIROBI | 8 | Y |
| 225 | 5 | NAKURU | 6 | NAKURU | NAIROBI | KIAMBU | NAIROBI | NAIROBI | NAIROBI | 19 | N |
| 241 | 5 | NAIROBI | 23 | NAIROBI | NAIROBI | NAIROBI | NAIROBI | NAIROBI | NAIROBI | 23 | N |

**Supplementary Table 4: Enrolled Volunteers in Groups 2, 4 and 6 (n=14)**

| **Subject** | **Group** | **Place of Birth (POB)** | **Years in POB** | **Location Childhood** | **Location Primary School** | **Location High School** | **Location Higher Education** | **Location Most Time Spent** | **Location 3 Years Prior to CHMI** | **Years in Nairobi Prior to CHMI** | **Time Spent Malaria Endemic Region** |
| --- | --- | --- | --- | --- | --- | --- | --- | --- | --- | --- | --- |
| 104 | 2 | MACHAKOS | 15 | MACHAKOS | MACHAKOS | MACHAKOS | NAIROBI | MACHAKOS | NAIROBI | 9 | Y |
| 110 | 2 | KAKAMEGA | 22 | KAKAMEGA | KAKAMEGA | KAKAMEGA | KAKAMEGA | KAKAMEGA | KAKAMEGA | 2 MONTHS | Y |
| 150 | 4 | NAIROBI | 6 | NAIROBI | NYANZA | NYANZA | N/A | NYANZA | NAIROBI | 5 | Y |
| 179 | 4 | KITALE | 14 | KITALE | KITALE | TESO, WESTERN | NAIROBI | KITALE | NAIROBI | 4 | Y |
| 105 | 6 | MWINGI | 17 | MWINGI | MWINGI | MWINGI | NAIROBI | MWINGI | NAIROBI | 4 | Y |
| 137 | 6 | NAKURU | CAN'T RECALL | ELDORET | ELDORET | ELDORET | ELDORET | ELDORET | ELDORET & NAIROBI | 2 | Y |
| 151 | 6 | KENDU BAY | 16 | KENDU BAY | KENDU BAY | NAIROBI & KENDU BAY | NAIROBI | KENDU BAY | NAIROBI | 10 | Y |
| 156 | 6 | ISIBANIA | 18 | ISIBANIA | MIGORI | MBITA | NAIROBI | ISIBANIA | NAIROBI & MIGORI | 2 | Y |
| 178 | 6 | MURANG'A | 25 | MURANG'A | MURANG'A | MURANG'A | NAIROBI | MURANG'A | NAIROBI | 4 | Y |
| 193 | 6 | SIAYA | 3 | SIAYA | NAIROBI | NAIROBI | NAIROBI | NAIROBI | NAIROBI | 19 | Y |
| 209 | 6 | KITALE | 14 | KITALE | KITALE | NAKURU | NAIROBI | KITALE | NAIROBI | 5 | Y |
| 213 | 6 | TAITA | 18 | TAITA | TAITA, MOMBASA | TAITA | NAIROBI | TAITA | NAIROBI | 10 | Y |
| 220 | 6 | MERU | 12 | MERU | MERU | MERU | NAIROBI | MERU | NAIROBI | 9 | Y |
| 229 | 6 | NAIROBI | 26 | NAIROBI | NAIROBI | MURANG'A | NAIROBI | NAIROBI | NAIROBI | 10 | Y |

**Supplementary Table 5: Safety Monitoring Committee Schedule of Meetings**

| Milestone | Documents to Be Reviewed | Date/Time of Email of Documents to be Reviewed | Timeline |
| --- | --- | --- | --- |
| Preparatory Meeting – Background Information | Review of initial clinical trial documentation | 1 month before meeting | February 2013 |
| Preparatory Meeting 2 | Schedule of safety review during challenge week | 1 week before meeting | Week beginning 29^th^ April 2013 |
| Enrolment of Cohort 1 (Groups 1 & 2) | Safety Data | Wednesday 8^th^ May 2013 0500 GMT | 48 hours post Enrolment of Cohort 1 |
| Enrolment of Cohort 2 (Groups 3 & 4) | Safety Data | Friday 10^th^ May 2013 0800 GMT | 48 hours post enrolment of Cohort 2 |
| Clinical Trial End | Safety & Infectivity Data | 1 month before meeting | November 2013 |

**Supplementary Table 6: Study Specific Clinical Standard Operating Procedures (SOPs)**

| SOP Number | SOP Title |
| --- | --- |
| SOP KC CLIN 001 | Recruitment & Screening for KEMRI Challenge Study |
| SOP KC CLIN 002 | Consent for KEMRI Challenge Study |
| SOP KC CLIN 003 | Sample Collection for Screening in KEMRI Challenge Study |
| SOP KC CLIN 004 | Blood Sample Collection and Handling for KEMRI Challenge Study |
| SOP KC CLIN 005 | Assessing Prior Exposure to *Plasmodium falciparum* |
| SOP KC CLIN 006 | Pre Enrolment Review for KEMRI Challenge Study |
| SOP KC CLIN 007 | Sample Collection for Pre Enrolment Review in KEMRI Challenge Study |
| SOP KC CLIN 008 | Administration of PfSPZ Challenge in KEMRI Challenge Study |
| SOP KC CLIN 009 | Clinical Reviews Post Challenge in KEMRI Challenge Study |
| SOP KC CLIN 010 | Sample Collection in Enrolled Volunteers in KEMRI Challenge Study |
| SOP KC CLIN 011 | Treatment for Malaria in KEMRI Challenge Study |
| SOP KC CLIN 012 | Transfer of Volunteers for Hospital Treatment in KEMRI Challenge Study |
| SOP KC CLIN 013 | Storage, Accountability & Dispensing of Medications & Products for KEMRI Challenge Study |
| SOP KC CLIN 014 | Discharge from CCR in KEMRI Challenge Study |
| SOP KC CLIN 015 | Payment of Volunteers in KEMRI Challenge Study |
| SOP KC CLIN 016 | Safety Reporting in KEMRI Challenge Study |
| SOP KC CLIN 017 | Assessing and Documenting Adverse Events in KEMRI Challenge Study |
| SOP KC CLIN 018 | Withdrawal of Volunteer from KEMRI Challenge Study |
| SOP KC CLIN 019 | Logistical Aspects of Inpatient Stay for KEMRI Challenge Study |
| SOP KC CLIN 020 | Safety Monitoring Committee reviews during KEMRI Challenge Study |
| SOP KC CLIN 021 | Sample and Results Management in KEMRI Challenge Study |
| SOP KC CLIN 022 | Study Forms and CRF Completion for KEMRI Challenge Study |
| SOP KC CLIN 023 | Delivering Screening Results to Volunteers in the KEMRI Challenge Study |

**Supplementary Table 7: Study Specific Laboratory Standard Operating Procedures (SOPs)**

| SOP Number | SOP Title |
| --- | --- |
| SOP KC LAB 001 | Whatman Filtering for PCR in KEMRI Challenge Study |
| SOP KC LAB 002 | Processing and Storage of Screening Serum Samples |
| SOP KC LAB 003 | Shipping of Samples from KEMRI CCR to Kilifi KEMRI in KEMRI Challenge Study |
| SOP KC LAB 004 | Processing of LiHep Samples for Exploratory Immunology in KEMRI Challenge |
| SOP KC LAB 005 | Processing and Storage of Serum Samples from Enrolled Volunteers |
| SOP KC LAB 006 | Processing and Storage of PAX Gene Tubes In KEMRI Challenge Study |
| SOP KC LAB 007 | Shipment of Immunology Samples to Oxford in KEMRI Challenge Study |
| SOP KC LAB 008 | KEMRI CCR Lab Preparations Relating to PfSPZ Challenge |
| SOP KC LAB 009 | Processing samples for HLA in KEMRI Challenge Study |
| SOP KC LAB 010 | Maintenance of Liquid Nitrogen Shipper for PfSPZ Challenge |
| SOP KC LAB 011 | Processing Samples for Parasite Genotyping |
| SOP KC LAB 012 | DNA extraction and PCR for Whatman Filtered Samples |
| SOP KC LAB 013 | Processing Samples for RNA PCR |
| SOP KC LAB 014 | Preparation of Blood Smears and Staining |
| SOP KC LAB 015 | Blood smear reading, quantification and interpretation |
| SOP KC LAB 016 | Preparation of Giemsa stock stain and Giemsa stain working solution |
| SOP KC LAB 017 | Buffer Solution Preparation for Blood Smears |
| SOP KC LAB 018 | Giemsa stain working solution QC |
| SOP KC LAB 019 | Malaria microscopy quality assurance |
| SOP KC LAB 020 | Blood smear slides storage and archiving |
| SOP KC LAB 021 | HIV test Algorithm for KEMRI Challenge Study |
| SOP KC LAB 022 | HIV testing – DETERMINE - for KEMRI Challenge Study |
| SOP KC LAB 023 | HIV testing – UNIGOLD - for KEMRI Challenge Study |
| SOP KC LAB 024 | Hepatitis B testing for KEMRI Challenge study |
| SOP KC LAB 025 | Hepatitis C testing for KEMRI Challenge study |
| SOP KC LAB 026 | Urinary pregnancy testing for KEMRI Challenge Study |
| SOP KC LAB 027 | Mission^®^ Dipstick Urinalysis in KEMRI Challenge Study |
| SOP KC LAB 028 | Preparation of QC Slides for KEMRI Malaria Challenge Study |
| SOP KC LAB 030 | Processing Samples for Haemoglobinopathy Screening in KEMRI Challenge Study |

**Supplementary Table 8: Feedback from Participants**

| **Did you understand the study?** | **What was your state of health on discharge?** | **What was the attitude of the nursing staff?** | **Were procedures appropriately explained to you?** | **Was the discharge process clearly explained?** | **What was your overall rating of participation in the study?**  **1-3 / 4-6 / 7-10** | **What areas could be improved?** |
| --- | --- | --- | --- | --- | --- | --- |
| Fully | Good | Excellent | Always | Clearly | 7 - 10 | Number of daily blood draws |
| Fully | Good | Excellent | Always | Clearly | 7 - 10 | - |
| Fully | Not yet improved | Good | Sometimes | Clearly | 7 - 10 | Improve phlebotomy skills |
| Fully | Improved | Good | Always | Clearly | 7 - 10 | Improve phlebotomy skills |
| Fully | Good | Excellent | Always | Clearly | 7 - 10 | - |
| Fully | Good | Excellent | Always | Clearly | 7 - 10 | Inpatient living facilities |
| Fully | Good | Good | Always | Clearly | 7 - 10 | Improve phlebotomy skills |
| Fully | Good | Good | Always | Clearly | 7 - 10 | Inpatient living facilities |
| Fully | Good | Good | Always | Clearly | 7 - 10 | Number of daily physical observations |
| Fully | Good | Excellent | Always | Clearly | 7 - 10 | Inpatient living facilities |
| Fully | Good | Excellent | Always | Clearly | 7 - 10 | Speed of discharge once criteria met |
| Fully | Good | Good | Sometimes | Clearly | 7 - 10 | Improve phlebotomy skills |
| Fully | Improved | Excellent | Always | Somehow clearly | 7 - 10 | Nursing care |
| Fully | Good | Excellent | Always | Clearly | 7 - 10 | Inpatient living facilities |
| Fully | Good | Excellent | Always | Clearly | 7 - 10 | - |
| Fully | Good | Excellent | Always | Clearly | 7 - 10 | - |
| Fully | Good | Good | Always | Clearly | 7 - 10 | - |
| Fully | Good | Excellent | Always | Clearly | 7 - 10 | - |
| Fully | Good | Excellent | Always | Clearly | 7 - 10 | - |
